# Carbonate system in the Cabo Frio upwelling

**DOI:** 10.1038/s41598-023-31479-x

**Published:** 2023-03-31

**Authors:** Carlos Augusto Ramos e Silva, Livia Viana de Godoy Fernandes, Flavo Elano Soares de Souza, Humberto Marotta, Flavio da Costa Fernandes, Thaise Machado Senez Mello, Nicole Silva Caliman Monteiro, Anderson Araújo Rocha, Ricardo Coutinho, Lohengrin Dias de Almeida Fernandes, Raimundo Nonato Damasceno, Ludmila Caetano dos Santos

**Affiliations:** 1 Postgraduate Program in Dynamics of Oceans and Earth, Federal Fluminense University, Niteroi, 24230-971 Brazil; 2 Department of Marine Biology, Federal Fluminense University, Niteroi, 59075-970 Brazil; 3 Center for Study of Water, Biomass, and Oil (NAB), Federal Fluminense University, Niterói, 24210-330 Brazil; 4 Agricultural School of Jundiaí, Federal University of Rio Grande do Norte, Macaíba, 59280-000 Brazil; 5 Sedimentary and Environmental Processes Laboratory (LAPSA), Federal Fluminense University, Niterói, 24210-346 Brazil; 6 Admiral Paulo Moreira Marine Research Institute, Arraial do Cabo, 28930-000 Brazil; 7 Ecosystems and Global Change Laboratory (LEMG), International Laboratory of Global Change (LINCGlobal), Niterói, Brazil

**Keywords:** Marine chemistry, Environmental monitoring, Carbon cycle

## Abstract

The quantitative assessment of the carbonate system represents one of the biggest challenges toward the "Sustainable Development Goals" defined by the United Nations in 2015. In this sense, the present study investigated the Spatio-temporal dynamics of the carbonate system and the effects of the *El Niño* and *La Niña* phenomena over the Cabo Frio upwelling area. The physical characterization of the site was carried out through data on wind speed and sea surface temperature. Water samples were also collected during the oceanographic cruise onboard the Diadorim R/V (Research Vessel). From these samples, the parameters of absolute and practical salinity, density, pH, total alkalinity, carbonate, calcite, aragonite, bicarbonate dissolved inorganic carbon, carbon dioxide, partial pressure of carbon, calcium, and total boron were obtained. The highest average concentration of bicarbonate in S1 (2018 µmol/kg) seems to contribute to the dissolved inorganic carbon values (2203 µmol/kg). The values of calcite saturation state, aragonite saturation state, and carbonate were higher on the surface of each station (calcite saturation state = 4.80–5.48; aragonite saturation state = 3.10–3.63, and carbonate = 189–216 µmol/kg). The mean values of pH were similar in the day/night samples (7.96/7.97). The whole carbonate system was calculated through thermodynamic modeling with the Marine Chemical Analysis (AQM) program loaded with the results of the following parameters: temperature, salinity, total alkalinity, and pH parameters. This manuscript presents original data on the carbonate system and the "acidification" process influenced by the Cabo Frio upwelling, which directly depends on the *El Niño* and *La Niña* phenomena oscillations in the sea surface temperature.

## Introduction

Carbon dioxide (CO_2_) sources, transport mechanisms, and transformations are essential in oceanography field studies^1,2^. Inorganic CO_2_ can exhibit significant spatial and temporal variability within the same water mass since the ocean content is dependent on processes such as the atmospheric exchange through sea surface and organic matter degradation (both autochthonous and allochthonous derived)^3^.

The reduction in seawater pH caused by the increase in CO_2_ in this compartment can lead to a decrease in marine carbonate (Reaction 1), a process also known as ocean acidification (OA)^4^. The coastal ocean waters are naturally subjected to daily, seasonal, and even annual pH variations amplified by OA^5^. Seawater's pH oscillation affects the carbonate system speciation by reducing the amounts of ${\text{CO}}_{3}^{2-}$ while increasing CO_2_ and ${\text{HCO}}_{3}^{-}$ contents, interfering in the natural processes of photosynthesis and calcification of marine organisms, thus creating negative ecological, social, and economic impacts^6^.

The reduction in the amount of ${\text{CO}}_{3}^{2-}$ available in the water will reduce the ocean's capacity to remove the CO_2_ released into the atmosphere by human activities. The absorption of H^+^ and CO_2_ by ${\text{CO}}_{3}^{2-}$ reduces the capacity of shallow waters to retain CO_2_. Several authors have linked the calcium saturation state (Ω) with reducing the calcification capacity of organisms linked to a drop in ${\text{CO}}_{3}^{2-}$ availability^5,7,8^. As ${\text{CO}}_{3}^{2-}$ concentration decreases in the seawater (Reaction 1), there is a reduction in the carbonate saturation state (Ω) (Eq. 1). The Ω has been implied in the reduction of calcification of marine organisms. The sea animals that present carbonate structures such as skeletons, shells, and spines are the most affected by OA^9,10^.

Reaction 1 $$ {\text{CO}}_{2}  + {\text{CO}}_{3}^{{2 - }}  + {\text{H}}_{2} {\text{O}} = 2{\text{HCO}}_{3}^{ - }  $$

1 $$ \Omega  = \left[ {Ca^{{2 + }} } \right]\frac{{\left[ {CO_{3}^{{2 - }} } \right]}}{{k_{{sp}} }};{\text{k}}_{{{\text{sp}}}}  = {\text{solubility}}\;{\text{product}} $$

The speciation and quantification of the carbonate system are considered challenges for the Sustainable Development Goals (SDG) defined in 2015 by the United Nations^11^ for the next nine years. One of these challenges is establishing an analytical protocol and implementing a monitoring program for OA. Data obtained in studies of OA (pH, TA, [${\text{HCO}}_{3}^{-}$], [${\text{CO}}_{3}^{2-}$], [CO_2_]_aq_, ρCO_2_, Ω_calc_, Ω_arag_) are also essential to validate regional and global models of CO_2_ flow between ocean and atmosphere interface. OA studies have presented little progress in this direction^1,12–15^. The main difficulties related to the implementation of an OA Monitoring Program in coastal and oceanic waters are (1) the absence of a database of the carbonate system, (2) the lack of a unified protocol for the determination of pH and total alkalinity (TA), (3) the non-disclosure of the polynomial precision of the carbonate system data, and (4) lack of an integrated and open access data repository on CO_2_ fluxes in coastal and ocean waters^16^.

An OA monitoring network requires keeping records of the principal chemical parameters (i.e., pH and total alkalinity), which allows, for example, the determination of the aragonite saturation state (Ω_Arag_) and a complete description of the carbonate system^14^. The suitable parameters for this purpose are defined through the balance of the reactions (2–6) that occur when CO_2_ dissolves in seawater^2,17^ as follows:

Reaction 2 $${\text{CO}}_{2\left(\text{g}\right)}={\text{CO}}_{2\left(\text{aq}\right)}$$

Reaction 3 $${\text{CO}}_{2\left(\text{aq}\right)}+{{\text{H}}_{2}\text{O}}_{\left(\text{l}\right)}={\text{H}}_{2}{\text{CO}}_{3\left(\text{aq}\right)}$$

Reaction 4 $${\text{H}}_{2}{\text{CO}}_{3\left(\text{aq}\right)}={\text{H}}_{\left(\text{aq}\right)}^{+}+{\text{HCO}}_{3\left(\text{aq}\right)}^{-}$$

Reaction 5 $${\text{HCO}}_{3\left(\text{aq}\right)}^{-}={\text{H}}_{\left(\text{aq}\right)}^{+}+{\text{CO}}_{3\left(\text{aq}\right)}^{2-}$$

Reaction 6 $${\text{CO}}_{3\left(\text{aq}\right)}^{2-}+{\text{Ca}}_{\left(\text{aq}\right)}^{2+}={\text{CaCO}}_{3\left(\text{s}\right)}$$

Climatic phenomena such as the El Niño Southern Oscillation can affect the intensity of upwelling by increasing the SST^18^. This is conditioned to the power of events that affect the tropical and subtropical cyclone and anticyclone systems, changing the intensity of the winds nearby the upwelling region and leading to an increase in the SST, decreasing the strength of the upwelling. The influence of these phenomena in the Cabo Frio upwelling region has not yet been fully understood, nor have their implications for the carbonate saturation state. This experiment is part of an extensive study on the feasibility of implementing a unified protocol in a monitoring program for the acidification of coastal and offshore areas of the Brazilian ocean, such as (1) upwelling sites, (2) coral reefs, (3) oceanic islands, and (4) oil platforms. The present investigation originally elucidates the space–time dynamics of the carbonate system in the Cabo Frio resurgence and assesses *La Niña* and *El Niño* in the resurgence phenomenon.

## Material and methods

### Study site

**Figure 1:**
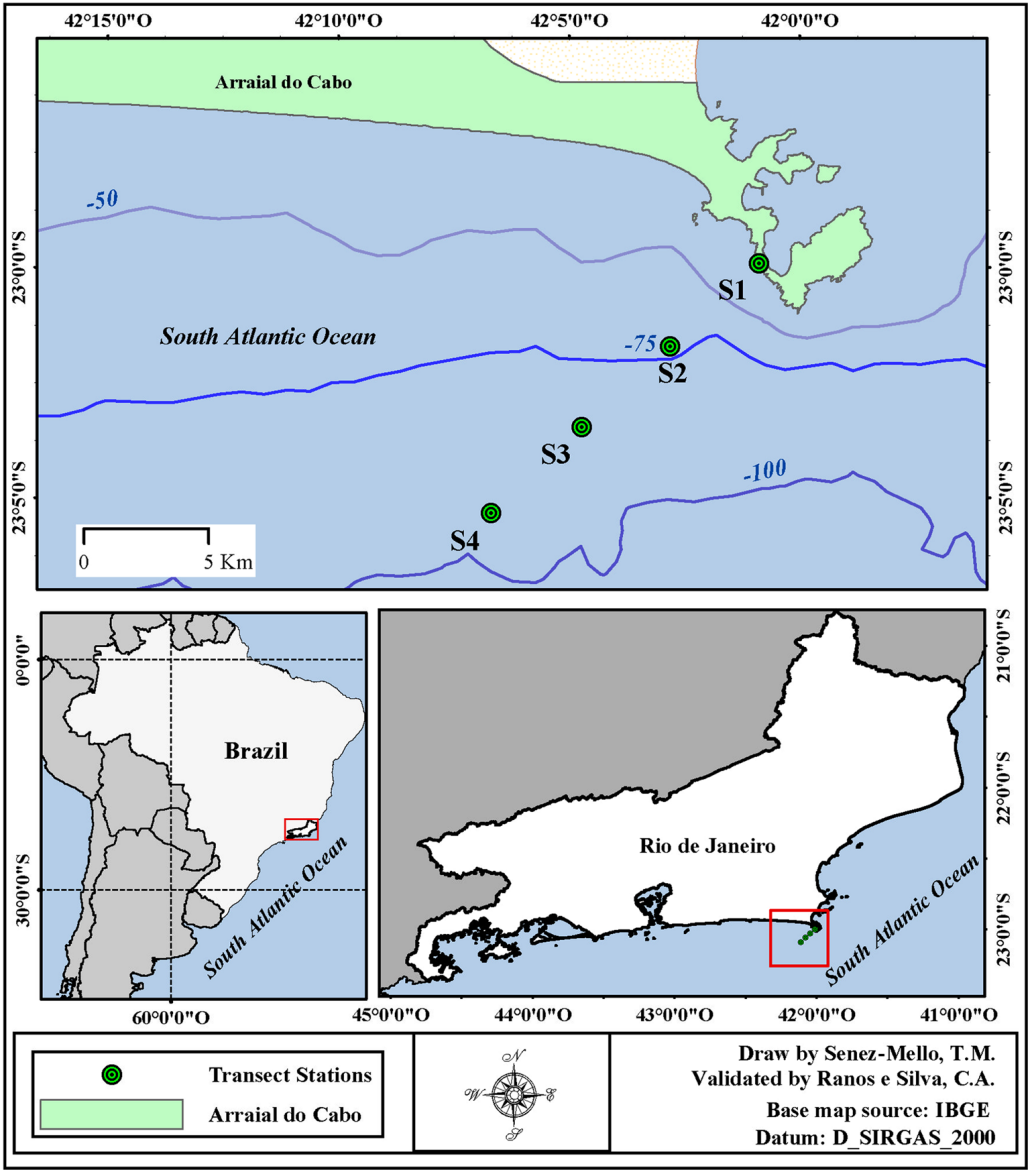
Study area. Sampling stations are represented by dots and respective numbers (S1, S2, S3, and S4). This map was generated with the ArcMap software v. 10.8.2 (https://www.esri.com/en-us/arcgis/about-arcgis/overview), by Senez-Mello, TM.

Located on the coast of Arraial do Cabo (Rio de Janeiro, Brazil, Fig. 1), the study site occurred between latitudes 22° 58′–23° 06′ South and longitudes 42° 10′–42° West. The Brazilian coast presents a NE-SW orientation in this region, causing the NE wind to blow parallel to the coast and the shallow coastal waters to move towards the open sea due to Ekman's transport^19^. Cabo Frio coastal upwelling phenomenon brings the deepest, coldest, and richest nutrient waters^20^.

Campos Basin is directly under the influence of the South Atlantic Subtropical Anticyclone (SASA)^21^, the main feature of the atmospheric circulation over the South Atlantic Ocean that affects the Brazilian weather and climate^22^. This feature is responsible for thermodynamic stability and low-intensity northeast winds that predominate in the southeast region of Brazil^21^. Teleconnection patterns (wave propagation in the upper atmosphere) can change the intensity and location of the South Atlantic subtropical high at any time of year, and changes in the positioning of the SASA generates a significant change in the wind pattern^23^.

Sun et al.^24^ analyzed data from 1979 to 2015 to compare the austral summer mean location of the SASA with the Southern Annular Mode (SAM)^25^ and extended multivariate *El Niño*–Southern Oscillation (ENSO) index (MEI)^26^. The authors found that when the SASA shifts poleward, the SAM was in a positive phase of *La Niña*, and when it shifts equatorward, the SAM was in a negative phase during *El Niño*. The wind speeds observed across Brazil can also describe this shift in the SASA pattern.

Three water masses occur at Arraial do Cabo from the shore to 500 m deep. The Tropical Water (TW) mass is found on the outer side of the continental shelf in the first 200 m and is characterized by a temperature range between 27.37 and 28.26 °C and a salinity range between 36.44 and 37.55 psu^1,27,28^. The South Atlantic Central Water (SACW) mass, ranging from 142 to 567 m in depth, is characterized by temperatures between 13.33 and 15.59 °C and salinity between 35.41 and 35.78 psu^1,20,27,29–31^. The SACW is a water mass rich in nutrients that reach the surface through the coastal Cabo Frio upwelling phenomenon^20,32^. The Coastal Water (CW) mass is found in the inner part of the continental shelf^33^, resulting from the mixture of continental waters, TW, and SACW. The CW is characterized by temperatures above 23 °C and salinity below 34 psu^34^. The primary water flux in the region is the Brazil Current System (BC). This current acts until approximately 500 m deep, carrying the TW and the SACW towards the south^31^.

The climate in the study area is classified as hot-semiarid^35^, characterized by intense regional evaporation and reduced rainfall if compared to adjacent areas, yielding an arid climate^36^.

### Wind velocity and surface temperature of the sea

Wind velocity and surface sea temperature (SST) data from the Cabo Frio (RJ) region, organized in a temporal series, were used to characterize the marine upwelling phenomenon. The daily averages of the wind velocity represented the NE (0°–45° direction) and SW (180°–270° direction) quadrants from September 2006 to December 2016. The data series of SST daily averages referred to the period between 1994 and 2016 (National Institute of Meteorology—www.inmet.gov.br). The data series of SST weekly averages referred to the period between 1994 and 2016 (data from the Admiral Paulo Moreira Marine Research Institute—IEAPM).

The daily average wind velocity from the NE and SW quadrants was tabulated with the respective SST (Sea Surface Temperature) averages from 2006 to 2016. In cases where the data of average wind velocity did not have corresponding SST data in the temporal series, the SST was estimated using the Kriging interpolation method. The estimate was obtained from the interpolation of the SST data in a regular grid, where: (X) corresponded to the time in days, (Y) to the time in years, and (Z) to the daily and annual SST^37^. A univariate statistic of residual values of SST resulting from the interpolation model was used to evaluate the standard error of the data, and to indicate the function that best fits the input data. The Q–Q (quantile–quantile) relation was used to analyze the wind velocity, NE, and SW, compared to its respective SST^38^. The data were compared to those of the *El Niño* and *La Niña* periods checking the influence of these events on wind velocity and SST^39^. Trend curves were also obtained relating the average wind velocity with the SST. Pearson's R^2^ coefficient was used to assess data correlation.

### Sampling design

The Diadorim R/V from the Brazilian Navy collected the water samples on January 20, 2016, using a Northeast/Southwest oriented transect. The first station (S1) was located between Cabo Frio Island and the continent, and the furthermost station (S4) was located 14 km from the coast, at the 100 m isobath (Table 1, Fig. 1).

**Table 1:** Geographic coordinates (datum WGS84), distance from the coast and depth of the stations sampled in this study (S1, S2, S3 and S4).

Stations	Coordinates (dd° mm′ ss.ss″)	Distance (km)	Depth (m)
S1	22° 59′ 54.75″ S 42°00′ 53.27″ W	0.130	20
S2	23° 01′ 43.05″ S 42° 02′ 49.22″ W	4.84	75
S3	23° 03′ 28.40″ S 42° 04′ 44.11″ W	9.46	85
S4	23° 05′ 20.56″ S 42° 06′ 42.17″ W	14.29	100

The sampling campaign involved a two-scale analysis. The spatial scale was held perpendicular to the coastline (S1–S4), Fig. 1. The ship was anchored in station 12 for 12 h for the temporal scale. Both campaigns were performed on the same day. In the spatial approach, water was sampled from the surface (~ 3 m) between 6:30 and 10:00 h (UTC) using a pump adapted to a hose without forming bubbles. The other water samples below the surface were collected through a Niskin bottle of 10 L for the middle (half of the total depth) and bottom (~ 5 m above the seafloor).

In the temporal approach, in station 2, the surface water samples were collected hourly (12 samples total), between 13:00 to 01:00 h (UTC), while middle and bottom water samples were collected in alternate hours: 13, 15, 17, 21, 23, and 1 h.

### Analytical methods

Water measurements, such as temperature, depth, and dissolved oxygen, were performed with a CTD vessel (Midas Valeport).

#### Thermodynamic modeling

The modeling and the procedural calibrations described in this manuscript were performed with the Marine Chemical Analysis (AQM) program^1,2,16,17^. The AQM is a package of thermodynamic equations, executed via MS Excel, which can predict the complex composition of the marine carbonate system. This package is based on measurements that can be relatively inexpensively (pH, temperature, and alkalinity), reducing the overall costs of ocean acidification monitoring programs. The AQM program is available upon request to the corresponding author’s email.

#### Statistical procedure

Non-parametric Kruskal Wallis test was chosen for comparisons between groups. All statistical tests were performed using the Statistica 7.0 software (TIBCO) with a significance level set at *p* < 0.05.

#### Analytical procedure

The analytical method was based on international procedures for studies involving the chemistry of inorganic carbon dioxide in marine waters^15,40,41^ with the necessary adaptations.

#### Total alkalinity (TA)

For the determination of TA, water samples were collected and filtered in a Nalgene filtration system through GF/F filters before being transferred to BOD type flasks (300 mL, Kimble brand) and immediately analyzed^40^.

The potentiometric determination was conducted with duplicate samples in an open thermostated glass cell, where 3 mL (to obtain v1) and 10 mL (to obtain v2) of HCl 0.1 M were added to each 100 mL sample^42^. The method consists in determining the slope of the line by obtaining two points for the function of Gran (F): F (1) defined by v1 and F (2) defined by v2^42^. A Thermo Scientific Orion Star potentiometer coupled to the Orion glass reference electrode cell, model 8102BNUWP was used for potentiometric determinations. The pH electrode was calibrated daily with "Tris" buffer (0.04 m) for sample readings (maximum 12 samples per day). Due to the reduced number of samples per day, the short period of the oceanographic cruise, and the constant working conditions (electricity source, solutions, and equipment), we chose to verify the electrode performance at the beginning and the end of the oceanographic cruise. The electrode's percent efficiency ranged between 99.49 and 99.54% concerning the theoretical Nernst value (59 mV). More details are available in hydrogen potential (pH).

The analytical precision and accuracy were calculated from five replicates of the reference material (Dickson–CRM, for oceanic CO_2_ measurements, batch 104)^43^, which obtained a 95% recovery rate from the expected value (Table 2). The calculated TA was obtained by the AQM program through the equation: TA (µmol/kg) = 660 + 47.6S, defined by Hunter^44^ for waters of the Atlantic and Pacific oceans by the GEOSECS Program. The normalized total alkalinity (NTA) was obtained by the AQM program using the equation: NTA (µmol/kg) = TA (µmol/kg) × 35/Salinity (g/kg), where 35 was assumed to be the representative salinity of the water masses.

**Table 2:** Total alkalinity measured from five replicates of the certified reference material (Dickson, oceanic CO_2_ measurements, batch 134).

	Total alkalinity
Expected value	2222.61 µmol/kg
Measured (mean)	2108.0 µmol/kg
Absolute error	− 115.0
Relative error	− 5.0
Variation coefficient	1.66%
Sample volume	50 L

#### Hydrogen potential (pH)

The total pH of the water samples collected during the cruise was determined in the "wet laboratory" as follows: pH_T_ (= − log([H^+^] + [(HSO_4_^−^]/c^o^), where c^o^ is the thermodynamic concentration (1 mol/kg-soln).

The internal solution of the combined pH electrode was filled up with 0.7 m NaCl to reduce the potential liquid junction. The electrode's electromotive force (emf) was related to the molar concentration of the proton [H^+^], as shown in Eq. (2).

2 $$E={E}^{o}-\left(\frac{RT}{F}\right)lnln \left[{H}^{+}\right]$$

where: E° is the standard electrode potential, determined by titrating a 0.7 m NaCl solution with 0.179 M HCl^45^. The pH_T_ (total scale) values were measured immediately after each collection at a constant temperature of 25 ℃ in a thermostatic cell connected to a microprocessed thermostatic bath with external circulation (Qimis) to avoid temperature bias^46^. The determinations were made by the Thermo Scientific Orion Star potentiometer coupled to the Orion glass reference electrode with a 0.7 m NaCl outer chamber filling solution, model 8102BNUWP. The analytical slope for the electrode was within ± 0.13 mV (theoretical Nernst value at 25 °C). The electrode was calibrated with a "Tris" buffer (0.04 m) prepared in the laboratory^47^, where pH values were assigned by spectrophotometry (m-cresol method)^16,40,48^. The "Tris" buffer allows the accuracy of 0.001 pH units units^47,49^. Subsequently, using the AQM program, the pH results were corrected for the temperature recorded at the sampling moment (pH_t_ = pH_25_ + A + Bt + Ct^2^)^50^.

#### Calcium (Ca) and total boron (TB)

The determination of Ca and TB was conducted using a MIP OES (microwave-induced plasma optical emission spectrometer, 4200 MP-AES, Agilent brand). The external analytical curves were made with monoelementary standards (1000 mg/L, VHG^®^) with concentrations ranging from 0.1 to 10 mg/L in an ultrapure water matrix. A matrix influence test was conducted in which it was found that both the boron and calcium signals did not show any significant difference between the ultrapure water matrices and the 500 mg/L NaCl solution. The calculated calcium and boric acid were also obtained using the equations: [Ca^2+^]_T_ = 2.938 × 10^−4^ × S^15^, and [B]_T_ = 0.000416 × (S/35)^51^. The analyzed versus calculated values of Ca and TB in water samples showed a relative error (RE%) from 0.2 to 8% (Table 3).

**Table 3:** Calcium and boron concentrations (µmol/L) in seawater at three depths: surface (s), middle (m), and bottom (b) at the four sampling stations (S1, S2, S3, and S4). The relative error (RE%) assumes the analyzed value as the expected result.

Station	B^a^ (µmol/L)	B^c^ (µmol/L)	RE (%) (µmol/L)	Ca^a^ (µmol/L)	Ca^c^ (µmol/L)	RE % (µmol/L)
S1s	412	411	**− 0.24**	11,073	10,618	**− 4.11**
S1m	419	412	**− 1.68**	11,091	10,584	**− 4.58**
S1b	425	411	**− 3.29**	11,126	10,777	**− 3.14**
S2s	403	411	**1.99**	11,073	10,990	**− 0.75**
S2m	410	411	**0.24**	11,231	10,857	**− 3.33**
S2b	425	412	**− 3.06**	11,423	10,614	**− 7.08**
S3s	408	410	**0.49**	11,248	10,954	**− 2.61**
S3m	412	411	**− 0.24**	11,300	10,772	**− 4.67**
S3b	418	412	**− 1.44**	11,493	10,545	**− 8.25**
S4s	406	410	**0.99**	11,266	10,842	**− 3.76**
S4m	413	411	**− 0.48**	11,353	10,938	**− 3.66**
S4b	422	412	**− 2.37**	11,580	10,792	**− 6.80**

*B*^*a*^ total analyzed boron, *B*^*c*^ total calculated boron (AQM Program), *Ca*^*a*^ total analyzed calcium, *Ca*^*c*^ total calculated calcium (AQM Program).

Significant values are in [bold].

#### Speciation and quantification of the carbonate system

All parameters from the inorganic CO_2_ system (CO_2_, ${\text{CO}}_{3}^{2-}$, ${\text{HCO}}_{3}^{-}$, DIC, ρCO_2_, Ω_Calc,_ and Ω_Arag_) were calculated using the carbonate system dissociation constant *K*^52^ defined as follows:

1. $${lnk}_{B}^{*}$$^53^
2. $${lnk}_{Si}^{*}$$^54^
3. $${lnk}_{1}^{*}$$(H_3_PO_4_)^55^
4. $${lnk}_{2}^{*}$$ ($${H}_{2}{PO}_{4}^{-}$$)^55^
5. $${lnk}_{3}^{*}$$ ($${HPO}_{4}^{2-}$$)^55^
6. $${lnk}_{2}^{*}$$ ($${CO}_{3}^{2-}$$)^56^

The aqueous concentrations (CO_2(aq)_) and the partial pressure (ρCO_2_) were calculated from the variables of temperature, salinity, pH, and TA and by using the thermodynamic and stoichiometric constant *K* (${pk}_{1}^{o}$, ${pk}_{2}^{o}$, ${pk}_{1}^{*}$, and ${pk}_{2}^{*}$)^57,58^. The AQM was also used in this phase, aiding the calculations.

#### Air-sea CO_2_ fluxes

The CO_2_ flux equation between oceans and the atmosphere is defined between aqueous CO_2_ and saturated CO_2_, defined as follows (Eq. 3):

3 $${F}_{{\text{CO}}_{2}}={k}_{T}\left[{\text{CO}}_{2water}-{\text{CO}}_{2saturated}\right]$$

Aqueous CO_2_ and saturated CO_2_ are components that characterize the "balance" of the flux equation (Eq. 3) determined in this study using the AQM program. Wind velocity has a significant effect on the gas transfer equation. The relationship between gas exchange and wind velocity can have non-linear effects on calculating the gas transfer velocities for particular wind velocity measurements, depending on the sampling design and the wind velocity^1^. The most critical parameter in the gases transfer velocity equation (Eq. 4) in terms of the function with wind velocity is based on the gaseous exchange coefficient (k_T_ in cm/h):

4 $${k}_{T}=0.31\times {u}^{2}\times \left[\frac{{S}_{c}}{660}\right]-0.5$$

where: u is the wind velocity module at 10 m from the surface in m/s, Sc is the Schmidt number of CO_2_ in seawater^59,60^, and 660 is the Sc value in seawater at 20 °C. The Schmidt number is defined as the water kinematic viscosity divided by a gas diffusion coefficient defined as follows (Eq. 5) by a polynomial:

5 $${S}_{C}=A-BT+C{T}^{2}-D{T}^{3}$$

The *k*_*T*_ calculation (Eq. 4) considers that the wind velocity (u) has a fundamental quadratic dependence on the CO_2_ flux calculation. Typically, the wind velocity sampling for the k_T_ calculation takes the climatological average into account at 10 m above the water surface^61^. However, such a procedure can include an error related to the average wind incompatibility and the CO_2_ sampling in situ*.* In addition, reading the wind speed on the sea surface (10 m) can produce an inaccurate result due to the orographic average of the wind induced by the vegetation. Thus, the closer the wind measurement is performed to the water surface, the more significant the effect of wind on Sc will be^2^.

## Results and discussion

### Spatial sampling

#### Temperature, salinity, and dissolved oxygen

**Figure 2:**
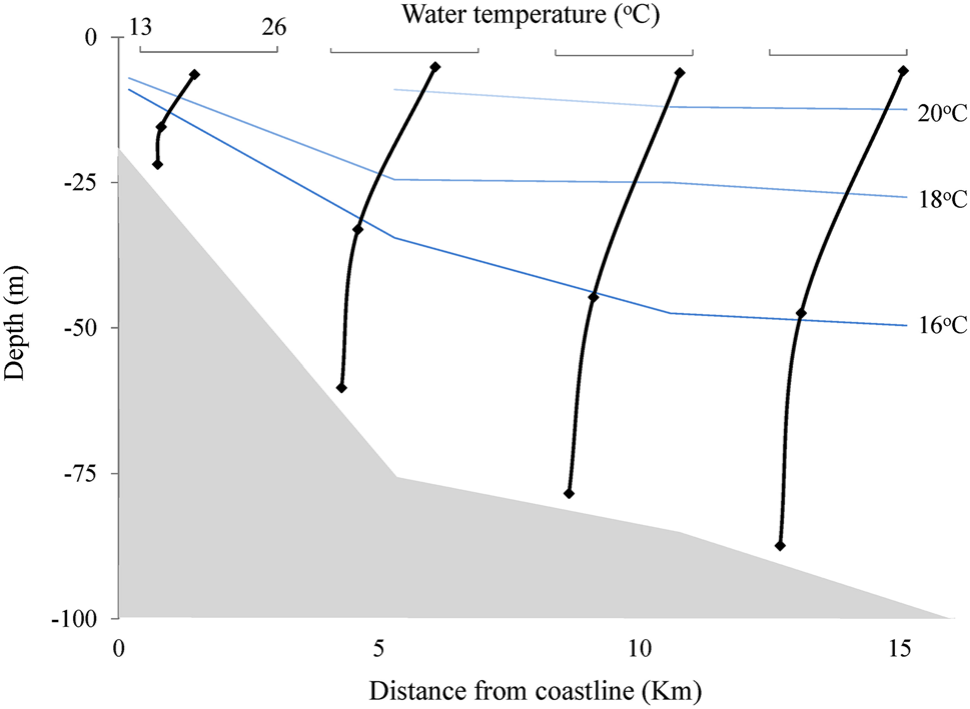
Temperature of the longitudinal samples from the surface, middle, and bottom of the water column.

**Figure 3:**
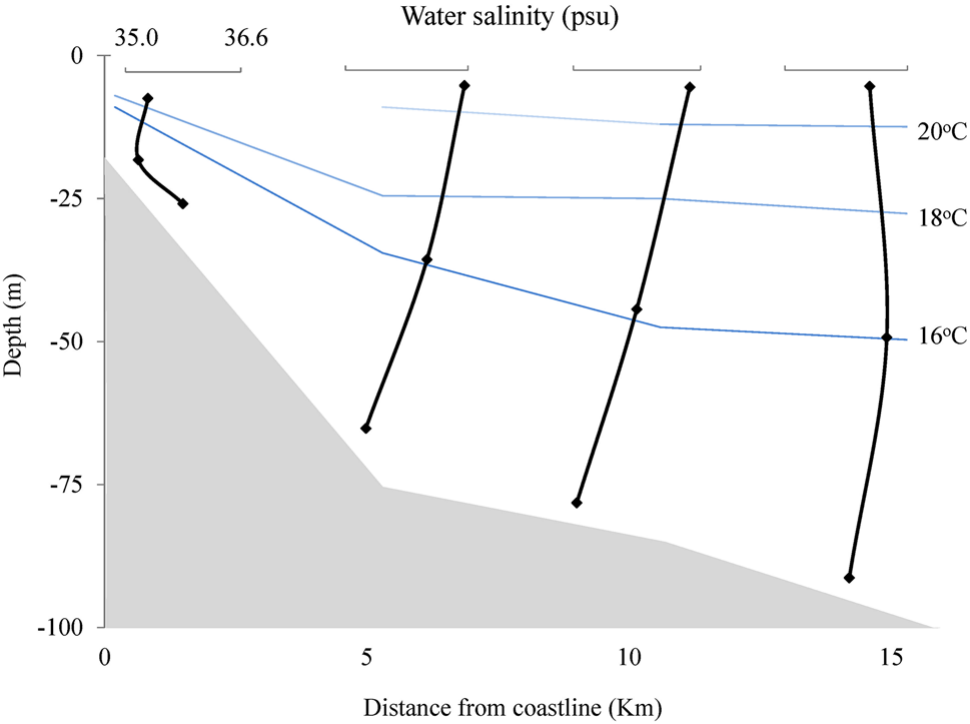
Salinity of the longitudinal samples from the surface, middle, and bottom of the water column.

**Figure 4:**
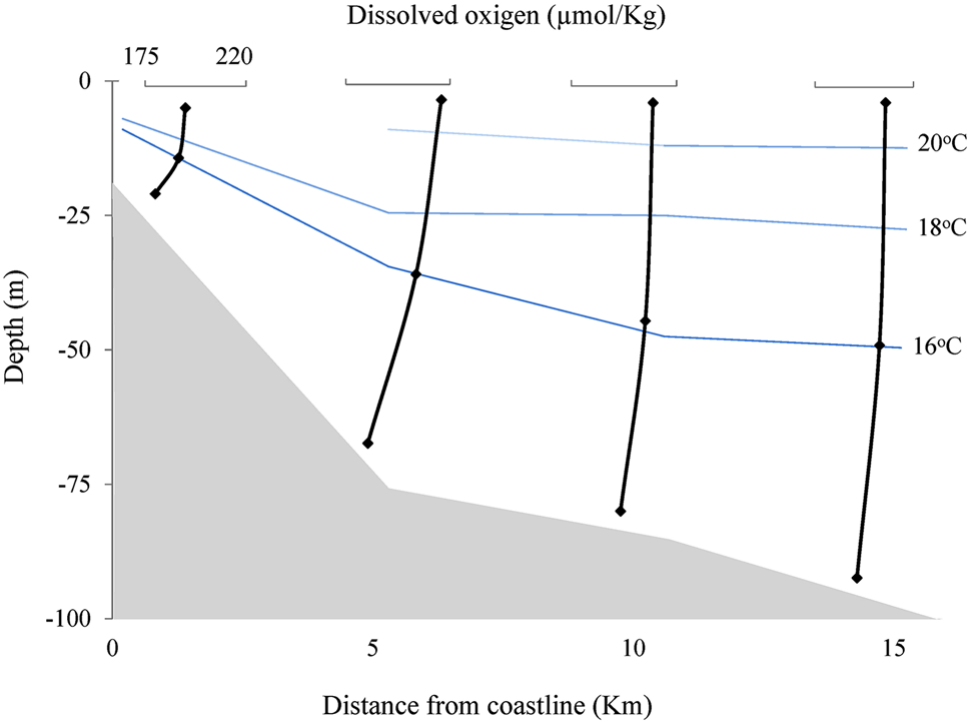
Dissolved oxygen in the longitudinal samples from the surface, middle, and bottom of the water column.

The physical–chemical data obtained at the four stations are represented in Figs. 2, 3, and 4. The lowest temperatures were recorded at the bottom of each station, varying from 13.9 °C (S2) to 14.7 °C (S1), showing the presence of SACW (colder and less saline waters). In station 1, the water emerged, showing lower temperatures and salinities, like the bottom samples from the other stations. On the surface of station 1, the temperature was 18.2 °C, and the salinity was 35.59 psu, where the temperature and salinity seem to characterize SACW^2^. The SACW was observed in S2, S3, and S4 at intermediate depths, where the temperature ranged from 15.4 to 16.0 °C and salinity between 35.55 and 35.74 psu. The presence of SACW was observed at the bottom of all stations. On the surface of S3–S4, water temperatures ranged from 22.2 to 25.7 °C, and salinities were greater than 36 psu. When increasing the distance from the coast (from S2–5 km), the temperature and salinity increased on the surface, characterizing the TW, see Figs. 2 and 3. Salinity did not present significant variations between stations and depths (Fig. 3). The highest values were recorded at each station surface, varying from 35.59 (S1) to 36.37 psu (S2), and the lowest values were recorded at the bottom, varying from 35.39 (S2) and 35.41 psu (S4).

All stations showed the highest DO concentrations (193–216 µmol/L) in superficial samples and the lowest concentrations (180–196 µmol/L) in bottom-most samples, Fig. 4. The highest DO contents were obtained in S2, S3, and S4, between 10 and 20 m depth, where respiration may predominate over productivity, making this water mass richer in organic and inorganic carbon and nutrients^20^. Thus, when SACW comes to the surface, it brings water rich in CO_2_ and nutrients and O_2_ depleted. In shallow water, the increase in oxygen solubility is favored by photosynthesis and gas exchange with the atmosphere (Fig. 4).

#### Wind dynamics and sea surface temperature (SST)

**Figure 5:**
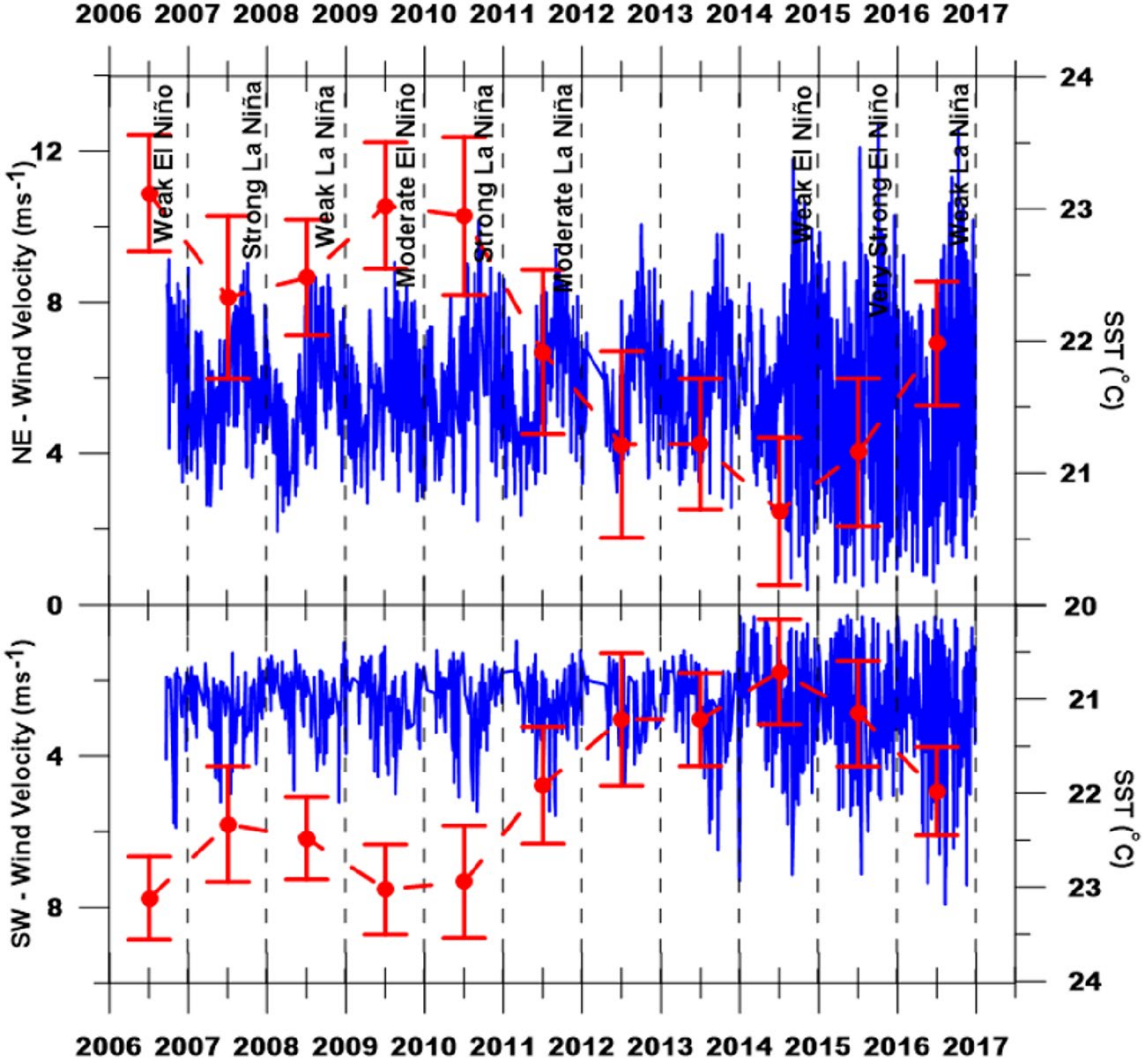
Wind velocity at quadrants SW and NE (blue line) with the respective variation of sea surface temperature (SST) at the Cabo Frio/RJ upwelling area (circle with a vertical bar in red), showing the average temperature and the standard deviation between 09/2006 and 12/2016, according to mild (Weak), and moderate to strong (Mod. to Str.) *El Niño* and *La Niña* events.

**Figure 6:**
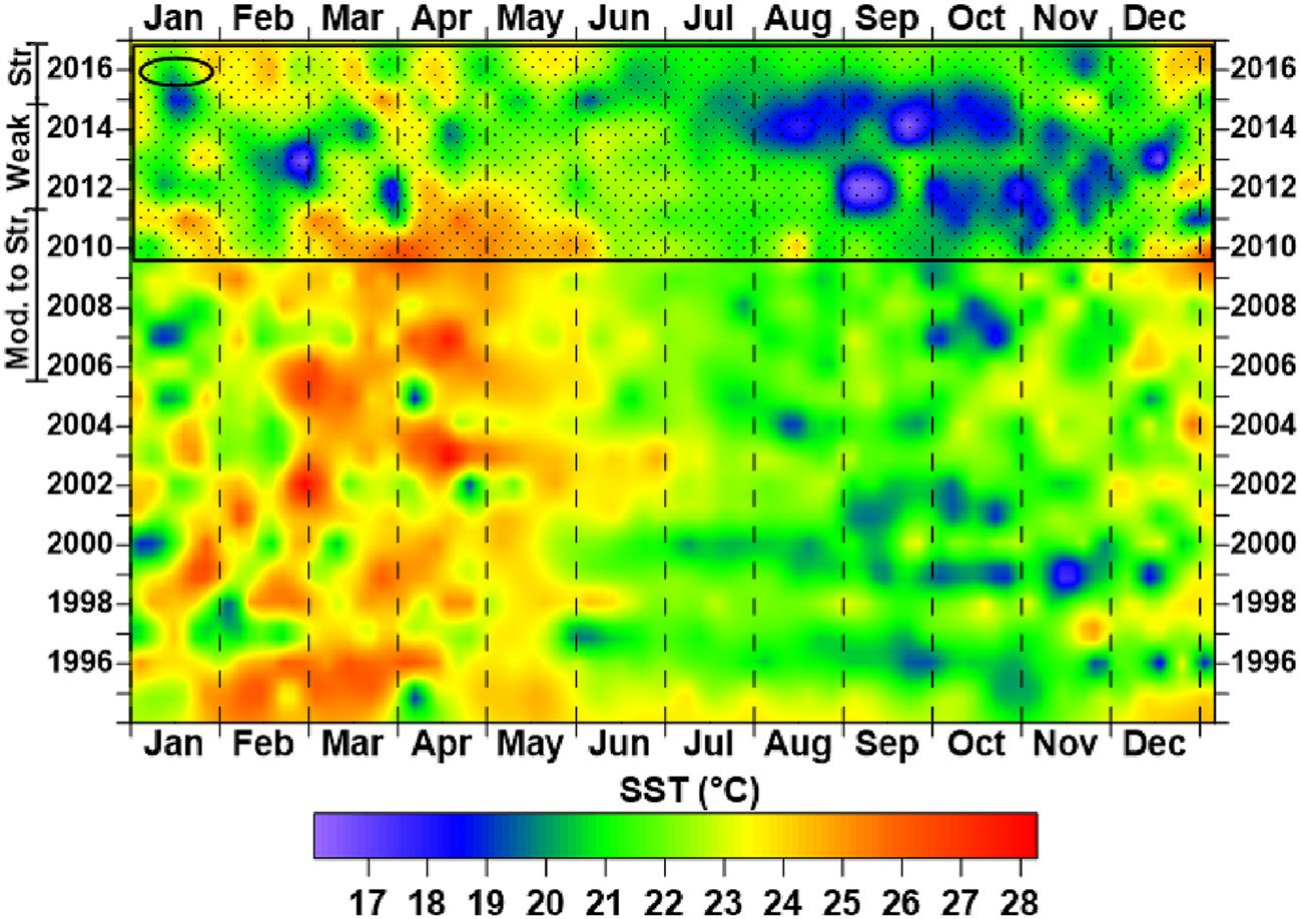
Temperature Grid of the Sea Surface Temperature (SST) at the Cabo Frio upwelling obtained by Kriging the SST data (1995–2016). Blue spots (cold): longer-term and intensity of the upwelling phenomenon. Red blots (hot): longer-term and intensity of temperature anomalies. Ellipse: SST in the period correspondent to the sampling campaign. Hachured rectangle: Str—increased wind magnitude period (from 2010 to 2016); Weak—absence of the events of a mild *El Niño* (from 2012 to 2013); and Mod. To Str—moderate to strong winds (from 2006 to 2010).

It was observed through a daily data series (from September 2006 to December 2016) of the study area that the NE and SW wind speeds increased from the year 2010. Between 2010 and 2015, this increase was simultaneous with the periods in which SST showed a downward trend favoring higher NE and SW wind speeds and a greater intensity of Cabo Frio's resurgence from 2010 to 2015. Between 2012 and 2013, the *El Niño* and *La Niña* events were observed, which favored the most considerable variations in SST given by the significant deviations from the mean, verified by the lowest SST concerning the other periods of the time series (Figs. 5 and 6).

In contrast, before 2010, moderate to strong *El Niño* and *La Niña* events affected the Cabo Frio resurgence phenomenon by reducing its intensity, increasing SST, and decreasing wind speed (Figs. 5 and 6). Elias^62^ had already observed a similar pattern in the Cabo Frio resurgence, but on a larger time scale as the *El Niño* periods, considering stronger winds responsible for changes in the resurgence process pattern, generating reflexes in the resurgence process local. The author also found that the Cabo Frio resurgence phenomenon is related to the frequency of the *El Niño* and *La Niña* events, causing changes in SST and the wind dynamics throughout the seasons. Thus, affecting the nutrient transport and dispersion from the sea bottom to the surface in the upwelling vicinity.

During the water sample collection period in January 2016, according to Figs. 5 and 6, sea surface temperatures were rising (on average from 20 to 22 °C) due to the strong *El Niño* that occurred in 2015. Also, a decrease in the average wind speed from 10 to 8 m/s was observed (Fig. 5). This decrease in wind speed corroborated with the increase in temperature of the water samples collected in the oceanographic campaign of January 2016 (22.7ºC average). This average temperature is even lower when compared to the average found between the years 2006 and 2010 (Figs. 5 and 6).

**Figure 7:**
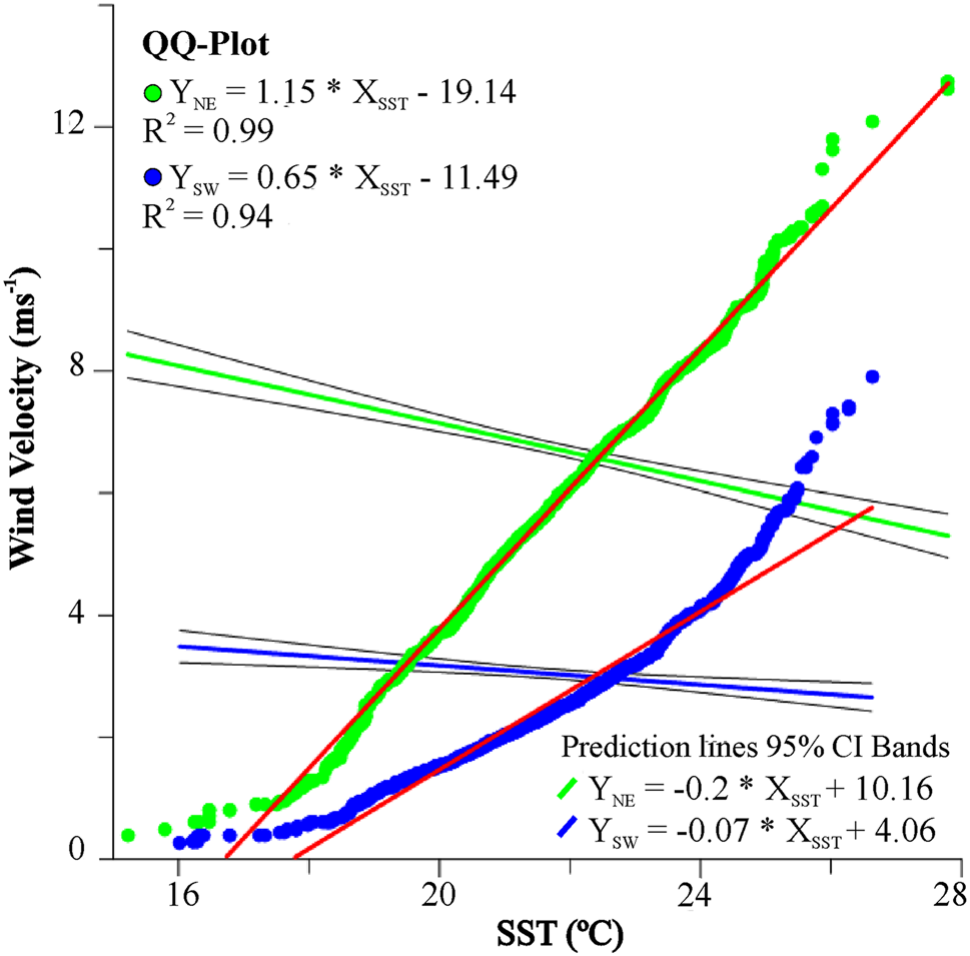
Quantile–Quantile plot (QQ-plot) displaying the SST and Wind Velocity data distribution from 2006 to 2016 in the Cabo Frio upwelling area. Blue dots: SW wind. Green dots: NE wind. Red lines: best-fit linear equation. Greenline: NE wind intensity trend curve; blue line: SW wind intensity trend curve, both fitted to 95% prediction bands.

When the *El Niño* and *La Niña* events are scarce or absent, it is observed that the SST decreases and the wind speeds up, increasing the intensity of the resurgence of Cabo Frio (Figs. 5 and 6). It was also observed that the duration of the more moderate events of *El Niño* and *La Niña* decreased the intensity of the resurgence, causing an increase in SST and a decrease in the speed of NE and SW winds (Figs. 5 and 6). This relationship between SST and wind speeds can be confirmed by comparing wind speed quantiles with SST in a Q–Q plot dispersion graph. This statistical test showed that the variables (wind speed x SST) have similar behavior in both wind directions (R^2^_NE_ = 0.99 and R^2^_SW_ = 0.94). In addition, the linear regression showed that the lower the SST, the greater the wind speed (Fig. 7).

The SST data and *El Niño* and *La Niña* presented in Fig. 6, which pointed to an influence on the wind pattern in the Cabo Frio upwelling area (Fig. 5), may be related to the movement of lunar nutation. The most evident effect is the water imprisonment in a nodal cycle or Saros in each hemisphere due to the alteration of the gravity center between the Moon and the Earth^63^.

This 18.6-year cycle coincides from 1993 to 2011, with a minimum lunar orbit declination (− 18.35°) in 1998 and the maximum (− 28.65°) in 2006. The minimum lunar declination occurred again in the following Saros cycle in 2016 (U.S. Naval Ephemeris Observatory). This observation coincided with the trapping of the warmest waters in the Southern Hemisphere, which may influence the decrease in the frequency of the Cabo Frio resurgence phenomenon and increase the effects of *El Niño* and *La Niña* on the resurgence by decreasing the winds. From 2010 onwards, the upwelling frequency became more intense, with a more significant decrease in SST causing the highest observed wind speeds due to the diminishing effects of *El Niño* and *La Niña* on the upwelling. Exceptionally, concerning the other years of the time series, from 2010 onwards, there was a more significant variation in SST (on average of − 3 °C) and wind speed (on average of + 4 m/s) (Fig. 5). Similar effects of these recent changes in upwelling systems have already been described^64^ to understand the aragonite saturation state in monsoon areas in Indonesia^65^.

#### Carbonate system

**Figure 8:**
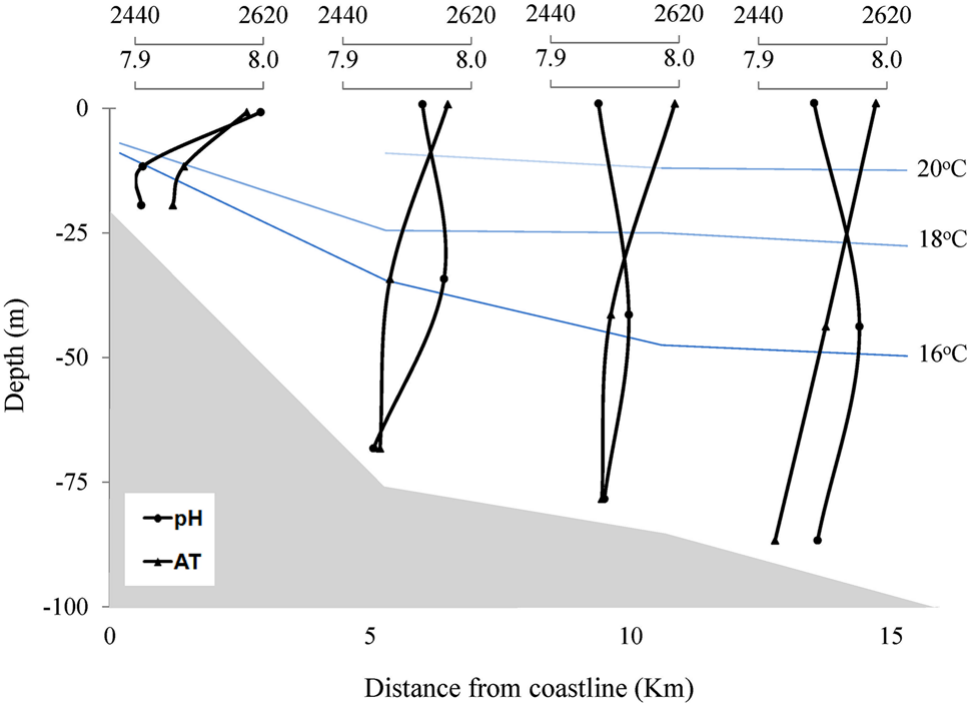
pH and Alkalinity of the longitudinal samples from the surface, middle, and bottom water column.

The Fig. 8 showed that pH did not have a pattern in the profiling of each station or between stations. The average value between stations ranged from 7.93 to 7.96. On the other hand, TA concentrations showed the highest surface values in all stations (2587–2614 µmol/kg), with the highest concentration in S3 (2614 µmol/kg). [CO_3_^2−^] seems to contribute significantly (R = 0.95; p < 0.05) to the TA values from the seasons.

The highest average concentration of ${\text{HCO}}_{3}^{-}$ in S1 (2018 µmol/kg), seems to contribute strongly (R = 0.92; p < 0.05) to the DIC values (2203 µmol/kg). Part of the [${\text{HCO}}_{3}^{-}$] in S1 can be explained by the increase in ρCO_2_ generated by the decomposition of organic matter of natural origin present in the resurgence of water^66,67^ (reactions 7–9).

Reaction 7 $$ {\text{CH}}_{2} {\text{O}} + {\text{O}}_{2} \to {\text{CO}}_{2} + {\text{H}}_{2} {\text{O}} $$

Reaction 8 $$ {\text{CO}}_{{2({\text{aq}})}} + {\text{H}}_{2} {\text{O}}_{{({\text{l}})}} \leftrightarrow {\text{H}}_{2} {\text{CO}}_{{3({\text{aq}})}} $$

Reaction 9 $$ {\text{H}}_{2} {\text{CO}}_{{3({\text{aq}})}} \leftrightarrow {\text{H}}^{ + }_{{({\text{aq}})}} + {\text{HCO}}_{{3({\text{aq}})}}^{ - } $$

**Figure 9:**
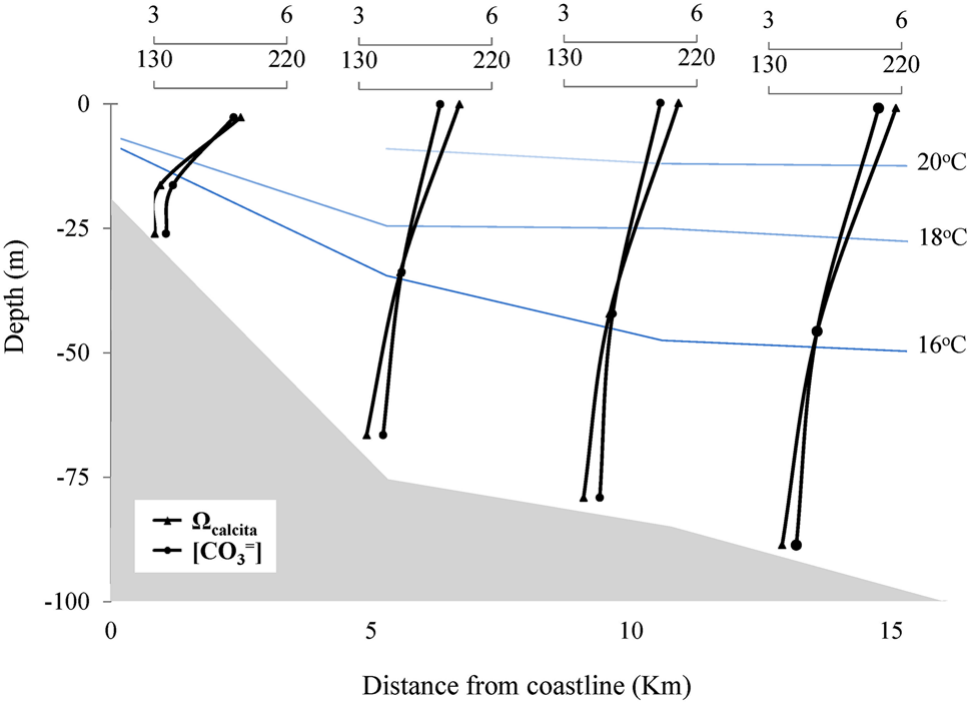
Calcite saturation state (Ω_Calcite_) and carbonate concentration (µmol/kg) of the longitudinal samples from the water column's surface, middle, and bottom.

SACW also contributes as a source of ${\text{HCO}}_{3}^{-}$, in S1 through the upwelling phenomenon. The values of Ω_Calcite_, Ω_Aragonite_, and ${\text{CO}}_{3}^{2-}$ were higher on the surface of each station (Ω_Calcite_ = 4.80–5.48; Ω_Aragonite_ = 3.10–3.63, and ${\text{CO}}_{3}^{2-}$ = 189–216 µmol/kg), Fig. 9. The mixture of water masses favored by the bathymetry seems to influence the high values of the carbonate system in surface waters. SACW, rich in nutrients compared to other water masses in the region^66^, provides a photosynthetic activity that affects the carbonate system equilibrium constants^2,17^, favoring the increase of the saturation state, of the concentrations of the carbonate and the TA. Equilibrium reactions 4 and 5 elucidate this process.

Recent research^68–70^ suggested that Ω from seawater does not control the rate of calcification (calcifying fluid). That is, we cannot merely link the availability of carbonate (affected by the decrease in pH values) to the rate of calcification. Until now, no ${\text{CO}}_{3}^{2-}$ transporter has been found in calcifying organisms (for example, coccolithophorids). Conversely, there is ample evidence of ${\text{HCO}}_{3}^{-}$ transporters. The bicarbonate within the calcifying fluid provides the formation of calcium carbonate (Reaction 6).

Reactions involving the formation of calcium carbonate are dependent on the electrochemical gradient (${H}^{+}$) between the marine environment and the organisms' tissue and suitable Ω (higher) in the cytoplasmic fluid. Several gaps still need to be clarified concerning the calcification of marine organisms^6,71,72^. The pH is a masterful measure for its participation in various chemical equilibrium reactions (proton concentration), through which accurate data on the speciation and quantification of the carbonate system can be obtained at reduced costs^1,13,73^.

The events of *El Niño* and *La Niña* can strongly affect the parameters of the carbonate system since these parameters are affected by SACW. The direct influence is the bicarbonate transport, while the indirect influence is the nutrient transport by the SACW stimulating the photosynthetic activity of the surface waters (reactions 4 and 5), removing the CO_2_ from the water. It is essential to highlight that in the sampling period (Jan/2016), there was an extreme *El Niño* event, affecting the upwelling phenomenon and favoring an increase in SST and a decrease in the speed of NE and SW winds. The opposite scenario occurred before 2010 when the SST averages were higher, and the wind speeds were lower than in the following years (Figs. 5, 6, and 7).

The values of the carbonate system discussed above showed that the studied area has a low corrosive effect, unlike the other upwelled waters generally presenting pH values below 7.80 and reduced carbonate saturation state (< 1.0)^1^. The low corrosive effect was probably due to the water masses mixture (SACW and TW) and the low-intensity upwelling by decreased NE wind speeds 5 days before the collection (Figs. 5 and 6), characterizing the effects of the intense *El Niño* on the Cabo Frio upwelling system.

### Temporal sampling

**Figure 10:**
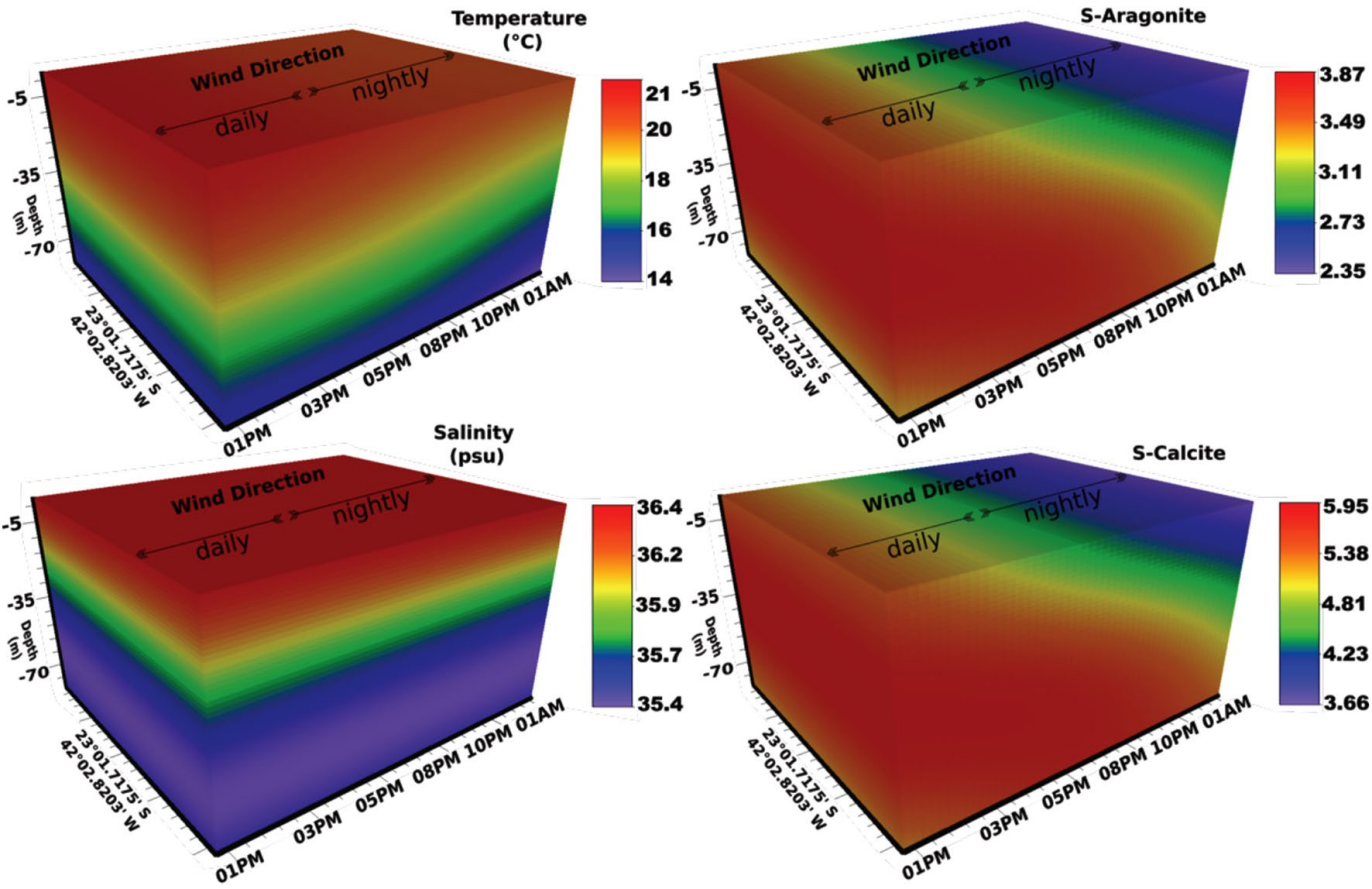
Temporal sampling. Temperature (°C), salinity (psu), aragonite, and calcite saturation state. X-axis: hour–day. Y-axis: coordinates of stations. Z-axis: Depth-m. Data sampled from 1:00 p.m. to 1:00 a.m. on the 20th and 21st of January 2016.

**Figure 11:**
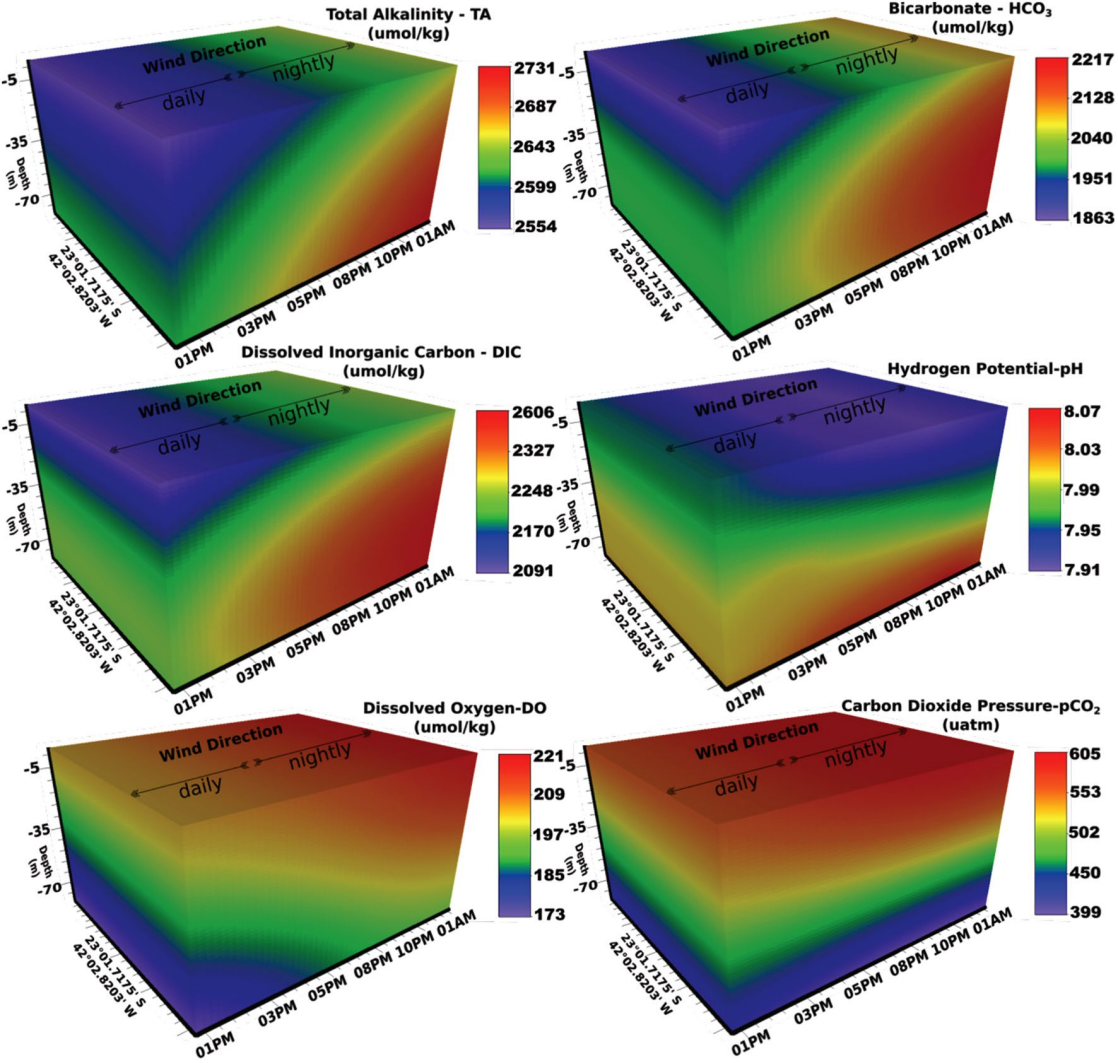
Temporal sampling. Total Alkalinity (µmol/kg), bicarbonate (µmol/kg), Dissolved Inorganic Carbon (µmol/kg), Potential Hydrogenionic (pH). Dissolved Oxygen (µmol/kg) and Carbon Dioxide Partial Pressure (atm). X-axis: hour–day. Y-axis: coordinates of stations. Z-axis: Depth-m. Data sampled from 1:00 p.m. to 1:00 a.m. on the 20th and 21st of January 2016.

In temporal sampling (S2), the effect of sunlight on the carbonate system parameters over 12 h was not elucidated (Figs. 10 and 11). The mean values of pH, TA, DIC, ρCO_2_, Ω_Ca_, and Ω_Ar_ were similar in the day/night samples, respectively: pH = 7.96/7.97; TA = 2.626/2.632 µmol/kg; DIC = 2.231/2.215 µmol/kg; ρCO_2_ = 523/518 µmol/kg; Ω_Ca_ = 4.6/4.6, and Ω_Ar_ = 3.0/3.0. In this time scale scenario, the physical variables (temperature and salinity) were determinants in the dynamics of the carbonate system's parameters concerning the biological processes (respiration and photosynthesis). It was also evident, through Fig. 10, that there was a mixture between the TW layers (temperature above 18 °C and salinity 36–37.37 psu) and SACW (temperature below 21 °C and salinity between 34.29 and 36.19 psu) over the sampling period.

The averages of the carbonate system parameters between the surface (TW) and bottom (SACW) layers were distinct, respectively: Ω_Ca_ = 5.2 and 3.9; Ω_Ar_ = 3.4 and 2.6; TA = 2642 and 2624 µmol/kg; ${\text{HCO}}_{3}^{-}$ = 2026 and 2217 µmol/kg; DIC = 2182 and 2284 µmol/kg; pH = 7.99 and 7.94; OD = 218 and 181 µmol/kg, and ρCO_2_ = 483 and 566 µmol/kg. The higher DIC values in SACW were due to the higher values of ρCO_2_ and HCO_3_^−^ (2026–2217 µmol/kg), already discussed in the carbonate system section. Reaction 1 explains the higher ${\text{HCO}}_{3}^{-}$ concentrations in this water mass. The raised ρCO_2_ found in SACW was caused by the decomposition of autochthonous organic matter in upwelled waters^66,67^ (reactions 7–9). Higher concentrations of ${\text{CO}}_{3}^{2-}$ on the surface (189–202 µmol/kg) seem to contribute to the higher values of the calcite and aragonite saturation state (Reaction 6) affected by the resurgence over 12 h. The higher values of the carbonate saturation state on the surface (TW) are favored by the photosynthetic activity that increases the concentration of carbonate ions (see Reaction 1).

The model used in Figs. 10 and 11 to spatialize the temperature and the carbonate system over the collection time (X) and depth (Y) clearly shows a mixture between TW and SACW water masses. Reduced upwelling intensity (20 to January 21, 2016) suggested the influence of the NE wind during the day. The lower intensity of the resurgence in the collection period from January 20 to January 21, 2016 (Fig. 6) suggests that the effects of the local NE wind blowing intensely towards the continent (*onshore*) during the day by a more significant temperature gradient between ocean and continent, can also promote the entrapment or stacking of surface water masses along the coast. On the other hand, with increasing water temperature, the winds blow towards the ocean (offshore) at night, accentuating the resurgence effect of SACW and its approach to the coast. This resurgence process during the night can be observed by the lower values of temperature and omega (Fig. 10). Furthermore, the higher TA, DIC, and [${\text{HCO}}_{3}^{-}$] values and pH decrease during the night endorse the effect of the upwelled SACW (Fig. 11).

The carbonate system of the present study, when compared to other study areas, showed a lower corrosive effect of SACW, probably due to the phenomena of *El Niño* and *La Ninã* over the Cabo Frio upwelling decreasing its intensity (see item 3.1.2). Studies^1^ of the carbonate system carried out in the South Atlantic (Ocean Basin Sergipe/Alagoas, Brazil) showed a more corrosive water from SACW (250 m deep), with the following values: pH = 7.74 mol/kg-sun; ρCO_2_ = 953 µmol/kg; Ω_Ca_ = 1.9; OD = 146 µmol/kg, and Ω_Ar_ = 1.2. Another study^65^ pointed to the effect of upwelling events on the aragonite saturation state, showing lower values (2.97–3.44) when compared to the area not affected by upwelling (4.45–3.57). An earlier study^5^ also described the same corrosive effect of upwelled acidified water on the continental shelf. All these authors suggested the corrosive effect of upwelled waters (pH < 7.75 and Ω_Ar_ < 1.0).

Recently, coastal areas have been attracting significant attention due to CO_2_ fluxes^74–77^. These researchers generated these CO_2_ fluxes as they were motivated by attempts to understand the effect CO_2_ has on ocean acidification, and coastal areas' role in CO_2_ sequestration. Irrespective of their motivations, the values generated by these CO_2_ fluxes are inaccurate in terms of the interface between water and air^2,61,78^. Indeed, fundamental physical K, such as the Schmidt number and the gaseous exchange coefficient (KT) components of the flux equation, are limited when considered in field experiments once they are empirically generated in controlled laboratory conditions. Such K do not consider other factors that interface with the CO_2_ flux, such as: water/air turbulence interface, air bubbles, surfactant substances, and precipitation. What is the associated error for estimating the flow with these physical factors that are not considered and the assumed laboratory conditions? The average flow of CO_2_ from water to the atmosphere in this work was 0.12 mmol/m^2^/day, which is relatively low when compared to the Caravelas estuary (5–1.377 mmol/m^2^/day)^16^, and average flow of CO_2_ found in upwelled waters off the coast of Chile (1.6–2.19 mmol/m^2^/dia)^79^.

This study presented new preliminary data on the dynamics of "acidification" in the Cabo Frio upwelling governed by the *El Niño* and *La Niña* phenomena, which control SST. Furthermore, it demonstrated the importance of implementing seasonal studies with protocols involving more sensitive analytical techniques with greater precision and reduced cost to generate more data and promote knowledge about the carbonate system along the Brazilian coast^1^.

## Conclusion

The El Niño Southern Oscillation might affect the upwelling intensity by raising the SST. This climate phenomenon influences the carbonate system since these parameters are affected by SACW. Calcium carbonate seems responsible for the increased values of TA in all studied stations. On the other hand, in the S1 station, DIC was influenced by bicarbonate concentrations from upwelled waters. In temporal sampling (S2), the effect of sunlight on the carbonate system parameters over 12 h was not observed.

Lastly, the results of our study emphasize the importance of ENOS phenomena, and the nodal cycle should be considered in studies of acidification of the oceans in resurgence areas. Interdisciplinary studies with the implementation of a specific protocol for temporal and seasonal scales are necessary to understand the effects on biota and support climate change models.

## Data Availability

The datasets used and/or analysed during the current study are available from the corresponding author on reasonable request.
